# Genetic encoding of complex traits

**DOI:** 10.1093/jxb/eraa498

**Published:** 2021-01-06

**Authors:** Stanislav Kopriva, Andreas P M Weber

**Affiliations:** 1 Institute for Plant Sciences and Cluster of Excellence on Plant Sciences (CEPLAS), University of Cologne, Cologne, Germany; 2 Institute of Plant Biochemistry, Cluster of Excellence on Plant Sciences (CEPLAS), Heinrich Heine University, Düsseldorf, Germany

**Keywords:** annual and perennial life history, C4 photosynthesis, complex traits, plant microbe interactions

**Plants provide food for humans, directly or through their use as feed for animals. However, arable land is becoming scarce and resources, including water and fertilizers, are becoming depleted under the effects of global climate change and the growing human population. To ensure food security for future generations, new approaches to improve crop productivity based on fundamental knowledge are needed. This special issue describes progress in our understanding of four traits that may be important for the development of future crops.**

Plants sustain life on earth by converting light energy, water, and CO_2_ into chemical energy and the building blocks of all organisms, including those that produce our food. However, a number of challenges threaten the production of sufficient food in the future, including loss of agricultural land, climate change, and the increasing human population. Furthermore, agriculture must address the United Nations Global Goals for Sustainable Development, in particular, the promotion of sustainable use of terrestrial ecosystems and ending hunger, achieving food security and improved nutrition, and promoting sustainable agriculture. To resolve the conflict between producing sufficient food for future generations and agricultural sustainability, it is necessary to develop innovative strategies for plant breeding and crop production. This is possible only through an increased fundamental understanding of how plants adapt to environmental conditions and constraints. The answers to these questions are encoded in the existing natural and artificially induced genetic variation that enables plant species and their associated microbes to colonize almost all terrestrial environments. However, many of the traits that are crucial for resource-efficient plant growth are complex, that is, they are encoded by multiple genes that interact with each other and with the environment. The contributions in this special issue address four such complex traits.

## Annual and perennial life history

Most perennial plants flower and reproduce repeatedly during their life cycle, whereas annuals reproduce only once. This divergence in life history is associated with many related phenotypic differences. For example, perennials often reproduce clonally, reducing their dependence on seed production, while annuals usually produce high numbers of seeds (Albani and Coupland, 2010). In addition, perennials are able to return to vegetative development after flowering and typically produce more extensive root systems, making perennial plants successful in marginal soils while at the same time providing beneficial ecosystem services (Bandaru *et al.*, 2013). The mechanisms underlying flowering control are thus of utmost importance for the annual and perennial life history (Kiefer *et al.*, 2017). As reviewed by Coupland *et al.* (2021), the key player in the control of flowering initiation is the MADS-domain transcription factor FLOWERING LOCUS C (FLC). FLC represses a transcriptional network of flowering genes, and is itself inhibited by cold, thus underlying the process of vernalization. In perennial plants, FLC suppresses post-vernalization flowering in axillary branches and thus ensures their return to vegetative development (Lazaro *et al.*, 2018). The review of Coupland *et al.* (2021) in this issue focuses on the gene regulatory networks controlled by FLC and explains how they influence floral transition. A part of the regulatory network of flowering initiation is FLOWERING LOCUS T (FLT), a small protein that is synthesized in the leaves and translocated via the phloem to the shoot apical meristem to induce floral transition (Corbesier *et al.*, 2007). Since grain yield in cereals is dependent on the control of reproductive development, understanding the mechanisms of control of flowering may contribute to the identification of novel breeding targets. Therefore, Pieper *et al.* (2021) analysed the function of *HvFT4*, one of 12 *FT-like* genes from barley. Overexpression of *HvFT4* led to delayed flowering of barley, caused by delayed spikelet initiation, and was also associated with reduced numbers of spikes and grains per spike. These and other results led to the conclusion that HvFT4 acts as a repressor of reproductive development in barley. Another piece of the jigsaw of control of reproductive development in barley is the identification of the photoperiod response gene *PHOTOPERIOD-H1* (*Ppd-H1*) as an integrator of photoperiod and drought stress signalling to modulate spike development (Gol *et al.*, 2021). While spring barley cultivars, which possess a mutation in the *Ppd-H1* gene, were affected in their floral development by drought, this effect was lost in lines with the original wild barley *Ppd-H1* allele. Thus, the results demonstrated that in barley, Ppd-H1 affects developmental plasticity in response to drought.

## C_4_ photosynthesis

C_4_ photosynthesis is an evolutionary innovation that results in significantly greater plant productivity due to more efficient carbon fixation and consequently also better water and nitrogen use efficiency. It is a complex trait that, despite requiring anatomical, biochemical, and physiological adaptations, evolved independently more than 60 times during land plant evolution (Sage, 2017). However, the major staple crops, with the exception of maize, are C_3_ plants, so engineering C_4_ photosynthesis in these crops presents an attractive possibility to improve future crop yields (Schuler *et al.*, 2016). Although such engineering—for example, of C_4_ rice—has been a long-standing goal, it still remains an enormous challenge to coordinately trigger alterations in plant metabolism and anatomy. Dissecting and understanding the genetic mechanisms underlying this complex trait are thus pre-requisites to exploit the C_4_ photosynthetic machinery to enhance the photosynthetic efficiency and productivity of C_3_ crops. A large number of tools and resources have been obtained within the past decade, including genome and transcriptome sequences from numerous C_4_ species (reviewed in Schlüter and Weber, 2020), a metabolic model of C_4_ photosynthesis (Bräutigam *et al.*, 2014), and mathematical models describing the evolution of C_4_ photosynthesis (Heckmann *et al.*, 2013; Mallmann *et al.*, 2014; Blätke and Bräutigam, 2019). In addition, attention is starting to be given to other areas of C_4_ biology, such as mineral nutrition (Jobe *et al.*, 2019). In this issue, Zamani-Nour *et al.* (2021) address another key issue for C_4_ photosynthesis, namely metabolite transport. Overexpression of chloroplastic 2-oxaloacetate/malate transporter (OMT1), which plays an important role in C_4_ plants, in rice unexpectedly led to large-scale alterations in metabolite accumulation, an imbalance between C and N metabolism, and a growth penalty. The phenotype could be reverted by overexpression of DiT2 transporter to export aspartate accumulated in the plastids. Thus, for engineering of the C_4_ photosynthetic cycle, broader C and N metabolite fluxes need to be considered.

## Molecular mechanisms of plant–microflora interactions

Plants in their natural environment are in constant interaction with a wide range of microorganisms. These interactions can be beneficial, neutral, or detrimental for the plant, depending on the taxonomic composition of the microbiota as well as the environment (Bulgarelli *et al.*, 2013). Interactions between plant roots and rhizosphere microbiota are, therefore, critical for plant fitness in natural environments as well as for agricultural crop yields. The past decade brought a great increase in understanding of the factors governing the composition of the plant microbiome and its benefits for plants (Haney *et al.*, 2015; Hiruma *et al.*, 2016; Muller *et al.*, 2016; Castrillo *et al.*, 2017; Finkel *et al.*, 2017; Jacoby and Kopriva, 2019). One of these factors is the glycans and glycoconjugates on the surface of the microbes, which are crucial for their recognition by plants. Wanke *et al.* (2021) review the role of these glycans in communication between plants and microbes. The focus of the review is the perception and recognition of the microbial extracellular glycans and their role in plant–microbe interactions. The leaf microbiome is the focus of a review by Chaudhry *et al.* (2021), which addresses the factors affecting the colonization of leaves and the microbe–microbe interactions within the leaf environment. The review discusses how microbial communities affect susceptibility to plant pathogens and vice versa, and the impact of pathogens and endophytes on leaf colonization. Particular attention is given to the plant immune system and its role in shaping the leaf microbiome.

## Role of metabolites in biotic interactions

Plants modulate the structure and function of microbial communities by exuding metabolites, which are an important carbon source for the growth of soil-borne microbes. But plants also produce a wide variety of secondary compounds, which play an active role in shaping the rhizosphere microbiome (Zhalnina *et al.*, 2018). Several metabolites have recently been shown to directly affect the composition or function of the microbial communities. In this issue, Jacoby *et al.* (2021) summarize current progress in the elucidation of the role of secondary metabolites in shaping the plant microbiome. A number of compounds, including coumarins, glucosinolates, benzoxazinoids, camalexin, and triterpenes, are discussed in detail, as are recent advances in methodologies that will be crucial for further dissecting the metabolic interdependence in the rhizosphere. One class of such secondary compounds, the glucosinolates, is the topic of a review by Mitreiter and Gigolashvili (2021). This review describes the molecular mechanisms of glucosinolate regulation, focusing on the integration of the glucosinolate transcriptional network with hormonal, environmental, and epigenetic factors. For the secondary metabolites to act in interaction with microbes, they need to be excreted from the cells. This transport, and particularly the G-family of ABC transporters, is reviewed by Gräfe and Schmitt (2021). The ABCG transporters are involved in various important processes, such as the response to pathogens, the formation of diffusion barriers, or the transport of phytohormones. However, the substrate specificity of many of these transporters is still unknown, and this review points out the importance of further research on these important transporters.

## References

[CIT0001] Albani MC, Coupland G 2010 Comparative analysis of flowering in annual and perennial plants. Current Topics in Developmental Biology 91, 323–348. 20705187 10.1016/S0070-2153(10)91011-9

[CIT0002] Bandaru V, Izaurralde RC, Manowitz D, Link R, Zhang X, Post WM 2013 Soil carbon change and net energy associated with biofuel production on marginal lands: a regional modeling perspective. Journal of Environmental Quality 42, 1802–1814. 25602420 10.2134/jeq2013.05.0171

[CIT0003] Blätke MA, Bräutigam A 2019 Evolution of C_4_ photosynthesis predicted by constraint-based modelling. eLife 8, e49305. 31799932 10.7554/eLife.49305 PMC6905489

[CIT0004] Bräutigam A, Schliesky S, Kulahoglu C, Osborne CP, Weber AP 2014 Towards an integrative model of C_4_ photosynthetic subtypes: insights from comparative transcriptome analysis of NAD-ME, NADP-ME, and PEP-CK C_4_ species. Journal of Experimental Botany 65, 3579–3593. 24642845 10.1093/jxb/eru100 PMC4085959

[CIT0005] Bulgarelli D, Schlaeppi K, Spaepen S, Ver Loren van Themaat E, Schulze-Lefert P 2013 Structure and functions of the bacterial microbiota of plants. Annual Review of Plant Biology, 64, 807–838. 10.1146/annurev-arplant-050312-120106 23373698

[CIT0006] Castrillo G, Teixeira PJ, Paredes SH, et al. 2017 Root microbiota drive direct integration of phosphate stress and immunity. Nature 543, 513–518. 28297714 10.1038/nature21417 PMC5364063

[CIT0007] Chaudhry V, Runge P, Sengupta P, Doehlemann G, Parker J, Kemen E 2021 Shaping the leaf microbiota: plant-microbe-microbe interactions. Journal of Experimental Botany 72, 36–56. 32910810 10.1093/jxb/eraa417 PMC8210630

[CIT0008] Corbesier L, Vincent C, Jang S, et al. 2007 FT protein movement contributes to long-distance signaling in floral induction of *Arabidopsis*. Science 316, 1030–1033. 17446353 10.1126/science.1141752

[CIT0009] Coupland G, Madrid E, Chandler JW 2021 Gene regulatory networks controlled by FLOWERING LOCUS C that confer variation in seasonal flowering and life history. Journal of Experimental Botany 72, 4–14. 32369593 10.1093/jxb/eraa216 PMC7816851

[CIT0010] Finkel OM, Castrillo G, Herrera Paredes S, Salas Gonzalez I, Dangl JL 2017 Understanding and exploiting plant beneficial microbes. Current Opinion in Plant Biology 38, 155–163. 28622659 10.1016/j.pbi.2017.04.018 PMC5561662

[CIT0011] Gol L, Haraldsson EB, von Korff Schmising M 2021 *Ppd-H1* integrates drought stress signals to control spike development and flowering time in barley. Journal of Experimental Botany 72, 122–136. 32459309 10.1093/jxb/eraa261 PMC7816852

[CIT0012] Gräfe K, Schmitt L 2021 The ABC transporter G subfamily in *Arabidopsis thaliana*. Journal of Experimental Botany 72, 92–106. 32459300 10.1093/jxb/eraa260

[CIT0013] Haney CH, Samuel BS, Bush J, Ausubel FM 2015 Associations with rhizosphere bacteria can confer an adaptive advantage to plants. Nature Plants 1, 15051. 27019743 10.1038/nplants.2015.51 PMC4806546

[CIT0014] Heckmann D, Schulze S, Denton A, Gowik U, Westhoff P, Weber AP, Lercher MJ 2013 Predicting C_4_ photosynthesis evolution: modular, individually adaptive steps on a Mount Fuji fitness landscape. Cell 153, 1579–1588. 23791184 10.1016/j.cell.2013.04.058

[CIT0015] Hiruma K, Gerlach N, Sacristan S, et al. 2016 Root endophyte *Colletotrichum tofieldiae* confers plant fitness benefits that are phosphate status dependent. Cell 165, 464–474. 26997485 10.1016/j.cell.2016.02.028 PMC4826447

[CIT0016] Jacoby RP, Kopriva S 2019 Metabolic niches in the rhizosphere microbiome: new tools and approaches to analyse metabolic mechanisms of plant–microbe nutrient exchange. Journal of Experimental Botany 70, 1087–1094. 30576534 10.1093/jxb/ery438

[CIT0017] Jacoby RP, Koprivova A, Kopriva S 2021 Pinpointing secondary metabolites that shape the composition and function of the plant microbiome. Journal of Experimental Botany 72, 57–69. 32995888 10.1093/jxb/eraa424 PMC7816845

[CIT0018] Jobe TO, Zenzen I, Rahimzadeh Karvansara P, Kopriva S 2019 Integration of sulfate assimilation with carbon and nitrogen metabolism in transition from C_3_ to C_4_ photosynthesis. Journal of Experimental Botany 70, 4211–4221. 31124557 10.1093/jxb/erz250 PMC6698703

[CIT0019] Kiefer C, Severing E, Karl R, Bergonzi S, Koch M, Tresch A, Coupland G 2017 Divergence of annual and perennial species in the Brassicaceae and the contribution of cis-acting variation at *FLC* orthologues. Molecular Ecology 26, 3437–3457. 28261921 10.1111/mec.14084 PMC5485006

[CIT0020] Lazaro A, Obeng-Hinneh E, Albani MC 2018 Extended vernalization regulates inflorescence fate in *Arabis alpina* by stably silencing *PERPETUAL FLOWERING1*. Plant Physiology 176, 2819–2833. 29467177 10.1104/pp.17.01754 PMC5884582

[CIT0021] Mallmann J, Heckmann D, Brautigam A, Lercher MJ, Weber AP, Westhoff P, Gowik U 2014 The role of photorespiration during the evolution of C_4_ photosynthesis in the genus *Flaveria*. eLife 3, e02478. 24935935 10.7554/eLife.02478 PMC4103682

[CIT0022] Mitreiter S, Gigolashvili T 2021 Regulation of glucosinolate biosynthesis. Journal of Experimental Botany 72, 70–91. 33313802 10.1093/jxb/eraa479

[CIT0023] Muller DB, Vogel C, Bai Y, Vorholt JA 2016 The plant microbiota: systems-level insights and perspectives. Annual Review of Genetics 50, 211–234. 10.1146/annurev-genet-120215-034952 27648643

[CIT0024] Pieper R, Tome F, Pankin A, von Korff Schmising M 2021 FLOWERING LOCUS T4 delays flowering and decreases fertility in barley. Journal of Experimental Botany 72, 107–121. 33048122 10.1093/jxb/eraa466 PMC7816854

[CIT0025] Sage RF 2017 A portrait of the C_4_ photosynthetic family on the 50th anniversary of its discovery: species number, evolutionary lineages, and Hall of Fame. Journal of Experimental Botany 68, 4039–4056. 28110278 10.1093/jxb/erx005

[CIT0026] Schuler ML, Mantegazza O, Weber AP 2016 Engineering C_4_ photosynthesis into C_3_ chassis in the synthetic biology age. The Plant Journal 87, 51–65. 26945781 10.1111/tpj.13155

[CIT0027] Schlüter U, Weber APM 2020 Regulation and evolution of C_4_ photosynthesis. Annual Review of Plant Biology 71, 183–215. 10.1146/annurev-arplant-042916-040915 32131603

[CIT0028] Wanke A, Malisic M, Wawra S, Zuccaro A 2021 Unraveling the sugar code: the role of microbial extracellular glycans in plant-microbe interactions. Journal of Experimental Botany 72, 15–35. 32929496 10.1093/jxb/eraa414 PMC7816849

[CIT0029] Zamani-Nour S, Lin H-C, Walker BJ, Mettler-Altmann T, Khoshravesh R, Karki S, Bagunu, ED, Sage TL, Quick P, Weber APM 2021 Overexpression of the chloroplastic 2-oxoglutarate/malate transporter in rice disturbs carbon and nitrogen homeostasis. Journal of Experimental Botany 72, 137–152. 32710115 10.1093/jxb/eraa343 PMC7816853

[CIT0030] Zhalnina K, Louie KB, Hao Z, et al. 2018 Dynamic root exudate chemistry and microbial substrate preferences drive patterns in rhizosphere microbial community assembly. Nature Microbiology 3, 470–480. 10.1038/s41564-018-0129-3 29556109

